# Nanoscale decoupling of electronic nematicity and structural anisotropy in FeSe thin films

**DOI:** 10.1038/s41467-020-20150-y

**Published:** 2021-01-04

**Authors:** Zheng Ren, Hong Li, He Zhao, Shrinkhala Sharma, Ziqiang Wang, Ilija Zeljkovic

**Affiliations:** Department of Physics, Boston College, 140 Commonwealth Ave, Chestnut Hill, MA 02467 USA

**Keywords:** Electronic properties and materials, Surfaces, interfaces and thin films

## Abstract

In a material prone to a nematic instability, anisotropic strain in principle provides a preferred symmetry-breaking direction for the electronic nematic state to follow. This is consistent with experimental observations, where electronic nematicity and structural anisotropy typically appear hand-in-hand. In this work, we discover that electronic nematicity can be locally decoupled from the underlying structural anisotropy in strain-engineered iron-selenide (FeSe) thin films. We use heteroepitaxial molecular beam epitaxy to grow FeSe with a nanoscale network of modulations that give rise to spatially varying strain. We map local anisotropic strain by analyzing scanning tunneling microscopy topographs, and visualize electronic nematic domains from concomitant spectroscopic maps. While the domains form so that the energy of nemato-elastic coupling is minimized, we observe distinct regions where electronic nematic ordering fails to flip direction, even though the underlying structural anisotropy is locally reversed. The findings point towards a nanometer-scale stiffness of the nematic order parameter.

## Abstract (web summary)

Here, in contrast to previous observations in Fe-based superconductors, scanning tunnelling microscopy of strain-patterned FeSe thin films reveals a local decoupling of electron nematicity and structural anisotropy, pointing towards a stiffness of the nematic order parameter at the nanoscale.

## Introduction

Electronic nematic ordering, characterized by breaking the rotational symmetry of the electronic structure, has emerged as a key signature of many unconventional superconductors^1–10^. In Fe-based superconductors, it is marked by a pronounced in-plane resistivity anisotropy^11–14^, lifting of the band degeneracy^15–18^ and directional scattering of electrons along a preferred Fe–Fe lattice vector^3,19–21^, typically accompanied by a small orthorhombic distortion along the same direction. To gain insight into the origin of electronic nematicity, experiments have explored its evolution with chemical composition^1,11–13,22^, temperature^10–19,22–24^, pressure^22^, and anisotropic strain^11–14,24,25^. Out of the array of these experimental handles, anisotropic strain presents a unique tuning knob that can controllably break the symmetry of the lattice. In turn, this can directly impact the overlap between inequivalent neighboring Fe–Fe orbitals, lifting the *d*_xz_ and *d*_yz_ orbital degeneracy, and in principle providing a preferred direction for the electronic nematicity to follow.

Microscopic imaging of Fe-based superconductors revealed the tendency of real materials to form electronic nematic domains, even in crystals under zero nominal strain^11,14,19,21,23,26–28^. The domains can have two orthogonal configurations oriented along inequivalent Fe–Fe lattice vectors. In bulk single crystals, the spatial extent of electronic nematic domains is typically at the order of a few micrometers^11,14,23,26,27^. In certain thin films however, the domain size is found to be significantly reduced compared to their bulk counterparts^21,28^. For example, while the domain size in FeSe single crystals is several micrometers^14,23^, it is reduced to ~10 nm length scales in thin films^21,28^. This possibly suggests that the substrate, which inevitably has a somewhat different lattice constant compared to the film, may play a role in the formation of smaller electronic nematic domains. However, quantitative measure of local symmetry-breaking strain in these systems, and its role in the development of nanoscale electronic nematic domains remains unexplored. In this work, we visualize the formation of electronic nematic domains around an underlying network of structural modulations in strained multilayer films of FeSe, and discover a de-coupling of the local antisymmetric strain and electronic nematic order.

## Results

### Observation of a network of structural modulations

**Fig. 1:**
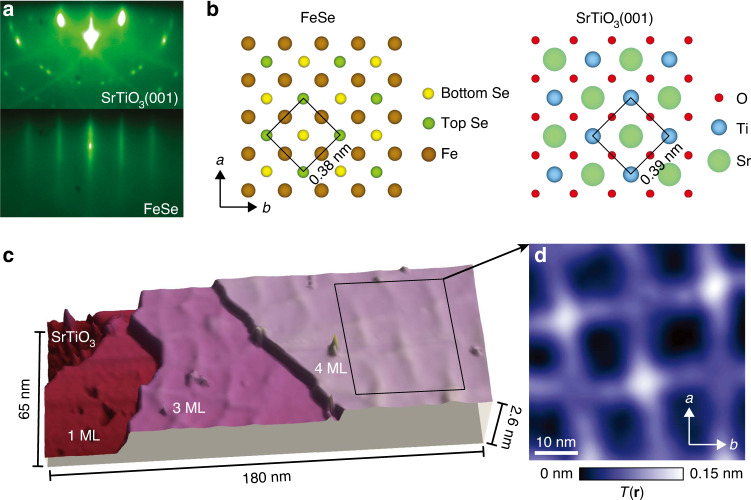
Structural characterization of FeSe thin films. **a** Reflection high-energy electron diffraction (RHEED) images (15 keV) of SrTiO_3_(001) (top) and FeSe (bottom). **b** Top view schematic of FeSe and SrTiO_3_ crystal structures, in which *a*-axis and *b*-axis denote the nearest-neighbor Fe–Fe directions. Black squares in **b** outline the Se (left) and Ti (right) unit cells. Brown, dark green, and yellow spheres in the FeSe crystal structure denote Fe, top Se, and bottom Se atoms, respectively. Light green, blue and red spheres in the SrTiO_3_ crystal structure denote Sr, Ti, and O atoms, respectively. **c** 3D rendered large-scale STM topograph that shows SrTiO_3_(001) substrate, 1 monolayer (ML) FeSe, 3 ML FeSe and 4 ML FeSe terraces. The periodic structural modulations can be more easily distinguished on the 3 ML and 4 ML FeSe layers. **d** Magnification of the region outlined by the black square in **c**, showing the structural modulations in the STM topograph *T*(**r**). Box smoothing over ~0.36 nm is applied to the topograph in **d**. STM setup conditions: **c** *I*_set_ = 10 pA, *V*_sample_ = 1 V; **d** *I*_set_ = 10 pA, *V*_sample_ = 600 mV.

FeSe presents an excellent playground to explore the interplay of electronic nematicity and symmetry breaking strain due to its structural simplicity and the absence of magnetic ordering that is present in many other Fe-based superconductors^29,30^. In principle, various experimental methods can be used to apply strain to a material, such as voltage-controlled piezoelectric setups^11,13,25,31,32^, mechanical actuators^27^, differential thermal contraction^24,33^, and heteroepitaxial film growth^34–37^. In this work, we use molecular beam epitaxy (MBE) to grow FeSe thin films (*a* = 3.8 Å) on SrTiO_3_(001), a substrate with a ~2% lattice mismatch (*a* = 3.9 Å) (Fig. 1a, b, see “Methods” section). We find a 2D network of modulations emerging at the surface of FeSe, propagating approximately along the Fe–Fe lattice directions (Fig. 1c, d). As we will subsequently show, this in turn leads to a spatially varying strain at the surface. The spatial distribution of modulation lines is qualitatively similar to those in heteroepitaxially-grown heterostructures of other chalcogenides^35–37^ and arsenides^34^. In our FeSe films, ranging from 3 to 6 monolayers in thickness, this distance between neighboring modulation lines is approximately 15–20 nm, consistent with the spacing determined from cross-sectional transmission electron microscopy^38^ and roughly consistent with the expected value based on the lattice constant mismatch between FeSe and SrTiO_3_(001) (Supplementary Note 1).

### Visualizing electronic nematic domains

**Fig. 2:**
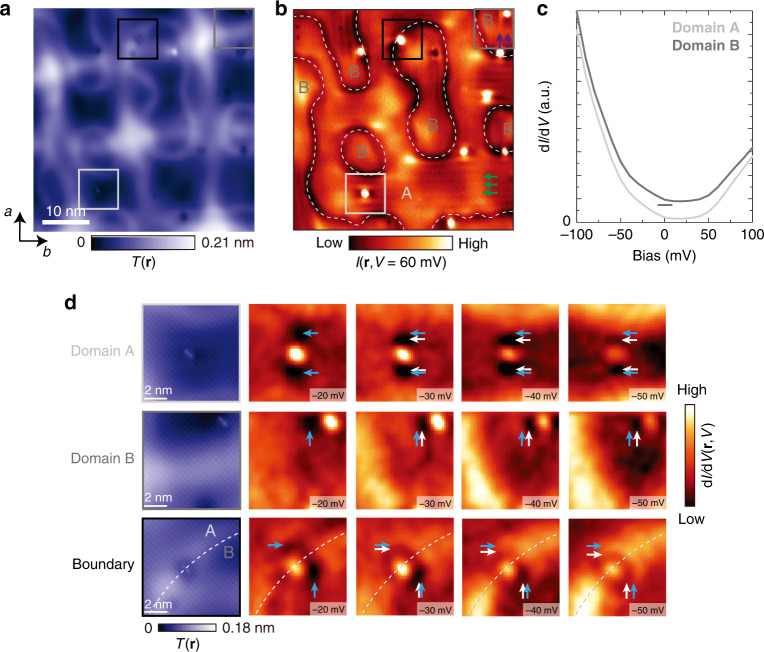
Visualizing electronic nematic domains. **a** Atomically-resolved STM topograph *T*(**r**), and **b** tunneling current map *I*(**r**,*V* = 60 mV) over an identical region, showing nematic domain boundaries denoted by white dashed lines. Horizontal charge-ordered stripes (denoted by green arrows) rotate into vertical stripes (denoted by purple arrows) across the boundaries between A and B. **c** Average differential conductance d*I*/d*V* spectra over nematic domains A and B, vertically offset for clarity. **d** Magnification of the regions near three different impurities in **a** outlined by the light gray, dark gray and black squares, which are located in the electronic nematic domain A, B and on the boundary, respectively. The last four figures in each row are d*I*/d*V(***r***,V)* maps encompassing each impurity, showing unidirectional dispersion from −20 to −50 mV. The blue and white arrows serve as guides to the eye for the dispersions. We note that the direction of this signal is not dependent on the impurity shape, as all three impurities shown are located at the equivalent Fe site. Symmetry of the electronic signal around impurities at the boundary of the two regions is broken down further, with one peak along each *a*-axis and *b*-axis, further supporting the intrinsic symmetry-broken electronic state of the system. STM setup conditions: **a** *I*_set_ = 110 pA, *V*_sample_ = −100 mV; **b** *I*_set_ = 110 pA, *V*_sample_ = −100 mV; **d** *I*_set_ = 110 pA, *V*_sample_ = −100 mV, *V*_exc_ = 5 mV.

**Fig. 3:**
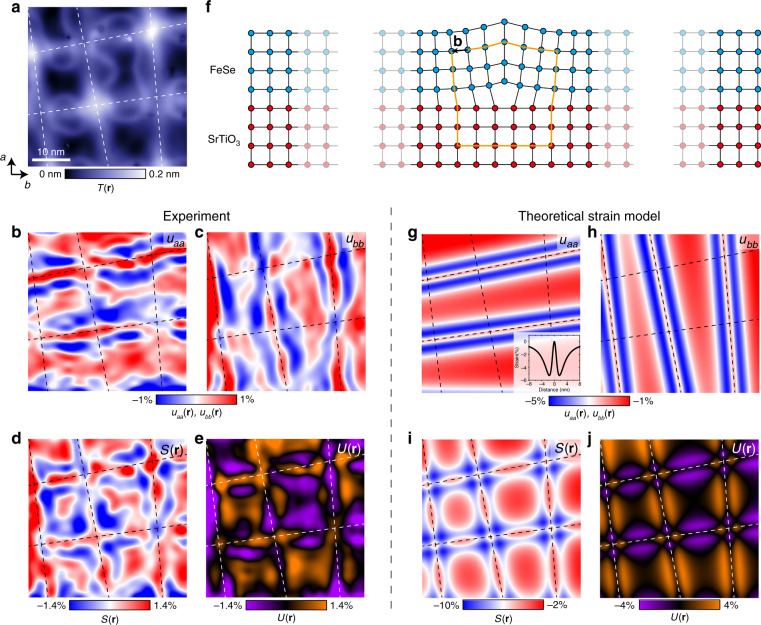
Strain analysis and comparison between experimental and theoretically predicted strain maps. **a** Atomically resolved STM topograph, where *a*-axis and *b*-axis denote the nearest-neighbor Fe–Fe directions. Dashed lines are guides to the eye for the structural modulations. Strain tensor components **b** $u_{{\mathrm{aa}}}\left( {\mathbf{r}} \right)$ and **c** $u_{{\mathrm{bb}}}\left( {\mathbf{r}} \right)$ derived from **a**. **d** Symmetric strain map $S\left( {\mathbf{r}} \right) \equiv u_{{\mathrm{aa}}}\left( {\mathbf{r}} \right) + u_{{\mathrm{bb}}}\left( {\mathbf{r}} \right)$. **e** Antisymmetric strain map $U\left( {\mathbf{r}} \right) \equiv u_{{\mathrm{aa}}}\left( {\mathbf{r}} \right) - u_{{\mathrm{bb}}}\left( {\mathbf{r}} \right)$. **f** Schematic of an edge dislocation. Vector **b** denotes the Burgers vector. Blue (red) circles schematically represent the FeSe (SrTiO_3_) lattice. Theoretical maps of **g** $u_{{\mathrm{aa}}}\left( {\mathbf{r}} \right)$, **h** $u_{{\mathrm{bb}}}\left( {\mathbf{r}} \right)$, **i** $S\left( {\mathbf{r}} \right)$ and **j** $U\left( {\mathbf{r}} \right)$. The inset in **g** shows the strain created by a single dislocation line as a function of the distance from it. In our model described in detail in Supplementary Note 7, Burgers vector **b** is set to be 0.53 nm, which is the Se–Se distance along [110] direction on the top or the bottom of an FeSe monolayer (ML), and the thickness *d* is set to be 1.6 nm, or 3 ML of FeSe. STM setup condition: **a** *I*_set_ = 110 pA, *V*_sample_ =−100 mV.

To characterize electronic properties of our thin films, we use low-temperature spectroscopic-imaging scanning tunneling microscopy. Although a single-unit-cell thick FeSe grown on SrTiO_3_(001) can exhibit superconductivity with ~10–15 meV pairing gap (Supplementary Fig. 7), the surface of thicker FeSe films (~a few to ~50 nm thick^21^) grown on the same substrate typically does not show superconducting behavior (Supplementary Note 8). By acquiring d*I*/d*V*(**r**,*V*) or *I*(**r**,*V*) spectra (where *I* is the tunneling current, *V* is the voltage applied to the sample, and **r** is the relative *xy*-position of the tip) on a densely-spaced pixel grid, we are able to visualize spatial variations in electronic density of states as a function of energy and position. We focus on an area shown in Fig. 1d, where we observe two striking features not immediately obvious from STM topographs (Fig. 2b). First, we can discern dark irregularly-shaped contours enclosing parts of the sample (denoted by white dashed lines). Second, we observe horizontal (an example denoted by green arrows) or vertical stripes (purple arrows) oriented along Fe–Fe lattice directions, with ~1.8 nm nearest-neighbor distance, which do not disperse as a function of energy in d*I*/d*V* maps (Supplementary Fig. 1). This has been interpreted as the formation of charge-stripes in the electronic nematic state^21,28^, with the direction of electronic nematicity rotating by 90° across the domain boundaries. This interpretation is further supported by dispersive C_2_-symmetric modulations pinned to individual dumbbell-shaped impurities, which also rotate by 90° across the same boundaries (Fig. 2d). Putting this information together, we conclude that the sample consists of two types of electronic nematic domains. We note that the smallest domains observed here are only ~100 nm^2^, significantly smaller than those in bulk single crystals^14,23^. As we will show, the reduced domain size can be attributed to the rapidly varying strain landscape (Fig. 3).

### Strain analysis

To measure local structural distortions, we start with an atomically-resolved STM topograph (Fig. 3a and Supplementary Fig. 3), and apply a geometric phase analysis method^33,37^ based on the Lawler–Fujita drift-correction algorithm^4^. This method allows us to determine the displacement of atoms ${\mathbf{u}}\left( {\mathbf{r}} \right) = u_{\mathrm{a}}\left( {\mathbf{r}} \right){\hat{\mathbf{a}}} + u_{\mathrm{b}}\left( {\mathbf{r}} \right){\hat{\mathbf{b}}}$ with picoscale resolution. The four-component strain tensor $u_{{\mathrm{ij}}}\left( {\mathbf{r}} \right) \equiv {\mathrm{d}}u_{\mathrm{i}}\left( {\mathbf{r}} \right)/{\mathrm{d}}r_{\mathrm{j}}$ (where *i*, *j* = *a*, *b*) can be used to extract different types of strain deformations. For example, $u_{{\mathrm{aa}}}\left( {\mathbf{r}} \right)$ represents the change of the lattice constant along the *a*-axis (relative to the average lattice constant in the field-of-view), with positive (negative) values denoting local tensile (compressive) strain. Taking into account strain along both lattice directions, it is convenient to define symmetric strain component: $S\left( {\mathbf{r}} \right) \equiv u_{{\mathrm{aa}}}\left( {\mathbf{r}} \right) + u_{{\mathrm{bb}}}\left( {\mathbf{r}} \right)$ and antisymmetric strain component: $U\left( {\mathbf{r}} \right) \equiv u_{{\mathrm{aa}}}\left( {\mathbf{r}} \right) - u_{{\mathrm{bb}}}\left( {\mathbf{r}} \right)$. The latter is particularly useful as a quantitative measure of structural anisotropy between the two lattice directions. We apply the strain analysis algorithm to the same area as in Fig. 2 to obtain strain maps (Fig. 3b–e). Tensile strain is observed along the modulation lines in both $u_{{\mathrm{aa}}}\left( {\mathbf{r}} \right)$ and $u_{{\mathrm{bb}}}\left( {\mathbf{r}} \right)$ maps, which is sandwiched by two ribbons of compressive strain. Further away from the modulation lines, there is tensile strain again in broader areas. To support the robustness of the strain algorithm, we note that strain maps calculated from STM topographs acquired in a range of different biases look qualitatively indistinguishable (Supplementary Figs. 2 and 4). Theoretically calculated^39,40^ strain maps based on a network of edge dislocations (Fig. 3g–j), Supplementary Note 7) also show a close resemblance to our experimental data (Fig. 3b–e). Therefore, we can conclude that the observed strain can be modeled well by a misfit dislocation network, with small differences that could be attributed to intrinsic orthorhombic distortion accompanying each electronic nematic domain.

### Correlation of nematic domains and antisymmetric strain

**Fig. 4:**
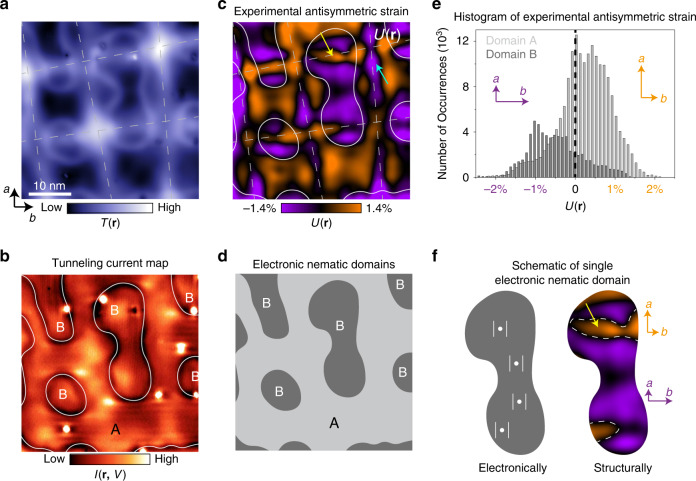
Interplay of electronic nematicity and local strain. **a** STM topograph *T*(**r**), and **b** tunneling current map *I*(**r**, *V* = 60 mV) acquired over an identical region of the sample. Dashed lines in **a**, **c** denote the network of structural modulations. Solid lines in **b**, **c** outline the electronic nematic domain boundaries. **c** The antisymmetric strain map *U*(**r**) calculated from **a**. **d** A schematic of electronic nematic domains determined from **b**, where A and B refer to orthogonal electronic nematic domains. **e** Histogram of the antisymmetric strain magnitude in electronic nematic domains A and B in **c**. **f** Magnification of a single nematic domain B and the antisymmetric strain distribution within it. White dots and double lines depict the Fe impurities and electronic stripes associated with the direction of electronic nematicity. Dashed lines in **f** denote the borders between regions of positive and negative antisymmetric strain. STM setup condition: **a** *I*_set_ = 110 pA, *V*_sample_ = −100 mV; **b** *I*_set_ = 110 pA, *V*_sample_ = −100 mV.

To investigate strain inhomogeneity further, we superimpose the outlines of electronic nematic domain boundaries on top of the antisymmetric strain map *U*(**r**) (Fig. 4c). For the electronic nematic domain A, where the charge-stripe wave vector is oriented along the *a*-axis, it is expected that the lattice constant along the *a*-axis (*a*_**0**_) is larger than that along the *b*-axis (*b*_**0**_)^21^. Indeed, the average antisymmetric strain within this region is consistent with this expectation (orange color in Fig. 4c). Similarly, within the electronic nematic domains B, where the charge-stripe wave vector propagates along the *b*-axis, we find that on average, *b*_**0**_ is greater than *a*_**0**_ (purple color in Fig. 4c). This is consistent with the global picture revealed in elasto-resistance experiments of bulk single crystals, where electronic nematic response followed the direction of externally applied anisotropic strain^11,12^. However, our ability to probe both local anisotropy and electronic nematicity at the nanoscale enables us to explore their correlation at previously inaccessible atomic length scales.

Interestingly, we find that the distribution of antisymmetric strain values within each electronic nematic domain is highly inhomogeneous (Fig. 4c, e). Moreover, not only do we find variations in magnitude, but also in the sign of the anisotropic strain. In other words, within electronic nematic domain A where the direction of electronic nematicity would dictate that *a*_0_ should be larger than *b*_0_, we observe sizeable regions (31% of the area) where this trend is opposite (an example denoted by blue arrow in Fig. 4c). The same observation is also apparent in the orthogonally oriented electronic nematic domain B (yellow arrow in Fig. 4c), where regions with *a*_0_ > *b*_0_ comprise 21% of the whole area. Thus, our experiments reveal a local decoupling of structural and electronic anisotropy in an electronic nematic system. We emphasize that this result does not rely on the theoretical strain model in Fig. 3 or the nature of strain modulation lines. The local strain on the surface is directly determined from atomically-resolved STM topographs, and then correlated with simultaneously acquired d*I*/d*V* maps where we can see electronic nematic domains.

## Discussion

To gain further insight into the electronic nematic domain distribution, we consider a phenomenological model where the electronic nematicity is described by an Ising order parameter field $\psi _{\mathrm{i}}$. In the simplest of terms, our system can be represented as a 2D square network of lattice sites, each characterized by an electronic nematic configuration $\psi _{\mathrm{i}}$ oriented along either *a* or *b*-axis. Antisymmetric strain $U({\mathbf{r}}_{\boldsymbol{i}})$ acts as an external field that linearly couples to $\psi _{\mathrm{i}}$, leading to an overall interaction energy $E_1 = - \alpha \mathop {\sum}\nolimits_{\mathrm{i}} {U({\mathbf{r}}_{\boldsymbol{i}})\psi _{\boldsymbol{i}}}$ $\left( {\alpha {\,}> {\,}0} \right)$, where index *i* runs over all lattice sites. If this was the only interaction in our system, to minimize the energy, the direction of $U({\mathbf{r}})$ would strictly dictate the orientation of $\psi _{\mathrm{i}}$ to be along the same direction. However, this is clearly contradictory to our observations (Fig. 4f). Therefore, we need to consider the correlation energy due to nearest neighbor interactions between the nematic fields: $E_2 = - \beta \mathop {\sum}\nolimits_{ < i,j > } {\psi _{\mathrm{i}}\psi _{\mathrm{j}}}$ $\left( {\beta \,> \, 0} \right)$. This term accounts for the increase in the overall energy along the boundary line, i.e., the domain wall, separating two orthogonally oriented electronic nematic domains, analogous to the energy increase due to anti-alignment of nearest neighbor spins in the ferromagnetic Ising model. This model suggests that competing contributions of *E*_1_ and *E*_2_ will contribute to the ultimate formation of domains.

It is important to notice that the relative magnitudes of *E*_1_ and *E*_2_ will strongly depend on the size of electronic nematic domains formed. For micron size domains in bulk single crystals, the number of nearest neighbor pairs along a domain boundary $\left( {N_{\mathrm{b}}} \right)$ is much smaller than the total number of sites $\left( {N_{{\mathrm{total}}}} \right)$. Correspondingly, *E*_2_ $\left( { \propto N_{\mathrm{b}}} \right)$ is negligible compared to *E*_1_ $\left( { \propto N_{{\mathrm{total}}}} \right)$. However, as the domain size decreases, $N_{\mathrm{b}}$ tends to $N_{{\mathrm{total}}}$, and *E*_2_ can become comparable to *E*_1_. This can explain why no electronic domains are formed along dashed white lines in Fig. 4f—the energy gain from aligning $\psi _{\mathrm{i}}$ with $U({\mathbf{r}})$ over the small area is simply not enough to overcome the energy loss from forming a nematic boundary. Therefore, the decoupling of electronic nematicity and structural anisotropy would be energetically favored.

Our experiments highlight an application of heteroepitaxy to create a densely spaced strain grid in thin films of FeSe. We reveal a direct evidence of local decoupling between electronic nematicity and structural anisotropy, which is likely a consequence of rapidly varying anisotropic strain. Given that antisymmetric strain in our films changes sign over only ~5 nanometers, but the smallest electronic nematic domains observed are several times larger than that, electronic nematic rigidity length scale is likely larger than ~5 nm. This in turn suggests that it may be difficult to partition the nematic domains beyond the size already achieved here. Future experiments tracking the domain distribution as a function of temperature, could shed light on any spatial variations of *T*_N_ in different strained regions and the robustness of domain boundaries with thermal cycling. Complementary to this, Te substitution for Se in strain-patterned FeSe can also allow explorations of domain formation by pushing the electronic nematic transition towards zero temperature^41^. In analogy to the magnetic field-driven motion of nematic domain boundaries detected in Ba(Fe_1−x_Co_x_)_2_As_2_^42^, which provided insight into substantial magneto-elastic coupling in that system, it would be interesting to investigate how the nanoscale nematic domains in FeSe behave in response to an in-plane magnetic field. Lastly, Potassium surface doping could lead to a re-emergence of superconductivity at the surface of our FeSe heterostructures^43,44^, and in turn enable studying the effects of spatially varying strain on superconductivity in FeSe-based compounds.

## Methods

### MBE growth

FeSe films were grown on Nb-doped (0.05 wt%) SrTiO_3_ (001) (Shinkosha). The substrates were sonicated in acetone and 2-propanol, followed by annealing in O_2_ supplied tube furnace at 1000 °C for 3 h. This step created √13 × √13 R33.7° surface reconstruction, which has been observed in both RHEED images and STM topographs (Supplementary Fig. 6). The substrates were then introduced into our MBE system (Fermion Instruments) with a base pressure of ~4 × 10^−10^ Torr. Continuously monitored by a pyrometer, the substrates were slowly heated up to ~400 °C for growth. Fe (99%) and Se (99.999%) were co-evaporated from two Knudsen cells held at 1100 and 145 °C, respectively, corresponding to flux rates of 9.87 × 10^−5^ atoms/(sec*Å^2^) for Fe and 3.37 × 10^−3^ atoms/(sec*Å^2^) for Se measured by the quartz crystal microbalance. At these relatively low flux rates, it takes about 28 min to form each monolayer, followed by post-growth annealing at ~450 °C for 2–3 h. After growth, the samples were either quickly transferred to the STM using a vacuum suitcase chamber held at ~1 × 10^−9^ Torr or capped with ~50 nm thick amorphous Se layer, and de-capped in the STM chamber at ~500 °C for 2 h. We note that FeSe films with modulations were observed in both films transferred by suitcase and de-capped thin films (Supplementary Note 4). We hypothesize that the modulations in multilayer FeSe may be related to the √13 × √13 R33.7° surface reconstruction of SrTiO_3_ (001)^38^.

### STM measurements

STM data was acquired using a Unisoku USM1300 STM at the base temperature of ~4.5 K. Spectroscopic measurements were made using a standard lock-in technique with 915 Hz frequency and bias excitation as detailed in figure captions. STM tips used were home-made chemically-etched tungsten tips, annealed in UHV to bright orange color prior to STM imaging.

## Supplementary information

Supplementary Information

Peer Review File

## Data Availability

Raw data used for the analysis shown in Figs. 2–4 can be downloaded from: 10.5281/zenodo.4273119.

## Code availability

The computer code used for data analysis is available upon request from the corresponding author.

## References

[1] Kasahara S (2012). Electronic nematicity above the structural and superconducting transition in BaFe_2_(A_1-x_P_x_)_2_. Nature.

[2] Vojta M (2009). Lattice symmetry breaking in cuprate superconductors: stripes, nematics, and superconductivity. Adv. Phys..

[3] Chuang T-M (2010). Nematic electronic structure in the ‘parent’ state of the iron-based superconductor Ca(Fe_1-x_Co_x_)_2_As_2_. Science.

[4] Lawler MJ (2010). Intra-unit-cell electronic nematicity of the high-*T*_*c*_ copper-oxide pseudogap states. Nature.

[5] Fang C, Yao H, Tsai W-F, Hu J, Kivelson SA (2008). Theory of electron nematic order in LaFeAsO. Phys. Rev. B.

[6] Xu C, Müller M, Sachdev S (2008). Ising and spin orders in the iron-based superconductors. Phys. Rev. B.

[7] Chubukov AV, Khodas M, Fernandes RM (2016). Magnetism, superconductivity, and spontaneous orbital order in iron-based superconductors: Which comes first and why?. Phys. Rev. X.

[8] Yamakawa Y, Onari S, Kontani H (2016). Nematicity and magnetism in FeSe and other families of Fe-based superconductors. Phys. Rev. X.

[9] Jiang K, Hu J, Ding H, Wang Z (2016). Interatomic Coulomb interaction and electron nematic bond order in FeSe. Phys. Rev. B.

[10] Baek S-H (2015). Orbital-driven nematicity in FeSe. Nat. Mater..

[11] Chu J-H (2010). In-plane resistivity anisotropy in an underdoped iron arsenide superconductor. Science.

[12] Chu J-H, Kuo H-H, Analytis JG, Fisher IR (2012). Divergent nematic susceptibility in an iron arsenide superconductor. Science.

[13] Kuo H-H, Chu J-H, Palmstrom JC, Kivelson SA, Fisher IR (2016). Ubiquitous signatures of nematic quantum criticality in optimally doped Fe-based superconductors. Science.

[14] Tanatar MA (2016). Origin of the resistivity anisotropy in the nematic phase of FeSe. Phys. Rev. Lett..

[15] Zhang P (2015). Observation of two distinct *d*_xz_/*d*_yz_ band splittings in FeSe. Phys. Rev. B.

[16] Shimojima T (2014). Lifting of xz/yz orbital degeneracy at the structural transition in detwinned FeSe. Phys. Rev. B.

[17] Nakayama K (2014). Reconstruction of band structure induced by electronic nematicity in an FeSe superconductor. Phys. Rev. Lett..

[18] Yi M (2019). Nematic energy scale and the missing electron pocket in FeSe. Phys. Rev. X.

[19] Rosenthal EP (2014). Visualization of electron nematicity and unidirectional antiferroic fluctuations at high temperatures in NaFeAs. Nat. Phys..

[20] Kostin A (2018). Imaging orbital-selective quasiparticles in the Hund’s metal state of FeSe. Nat. Mater..

[21] Li W (2017). Stripes developed at the strong limit of nematicity in FeSe film. Nat. Phys..

[22] Matsuura K (2017). Maximizing *T*_*c*_ by tuning nematicity and magnetism in FeSe_1−x_S_x_ superconductors. Nat. Commun..

[23] Rhodes LC, Watson MD, Haghighirad AA, Evtushinsky DV, Kim TK (2020). Revealing the single electron pocket of FeSe in a single orthorhombic domain. Phys. Rev. B.

[24] He M (2017). Dichotomy between in-plane magnetic susceptibility and resistivity anisotropies in extremely strained BaFe_2_As_2_. Nat. Commun..

[25] Andrade, E. F. et al. Visualizing the nonlinear coupling between strain and electronic nematicity in the iron pnictides by elasto-scanning tunneling spectroscopy. https://arxiv.org/abs/1812.05287 (2018).

[26] Tanatar MA (2009). Direct imaging of the structural domains in the iron pnictides AFe_2_As_2_ (A=Ca,Sr,Ba). Phys. Rev. B.

[27] Tanatar MA (2010). Uniaxial-strain mechanical detwinning of CaFe_2_As_2_ and BaFe_2_As_2_ crystals: optical and transport study. Phys. Rev. B.

[28] Yuan Y (2018). Edge states at nematic domain walls in FeSe films. Nano Lett..

[29] Böhmer AE, Kreisel A (2018). Nematicity, magnetism and superconductivity in FeSe. J. Phys. Condens. Matter.

[30] Coldea AI, Watson MD (2018). The key ingredients of the electronic structure of FeSe. Annu. Rev. Condens. Matter Phys..

[31] Hicks CW (2014). Strong increase of *T*_c_ of Sr_2_RuO_4_ under both tensile and compressive strain. Science.

[32] Mutch J (2019). Evidence for a strain-tuned topological phase transition in ZrTe_5_. Sci. Adv..

[33] Gao S (2018). Atomic-scale strain manipulation of a charge density wave. Proc. Natl Acad. Sci..

[34] Jain SC, Willander M, Maes H (1996). Stresses and strains in epilayers, stripes and quantum structures of III–V compound semiconductors. Semicond. Sci. Technol..

[35] Zeljkovic I (2015). Strain engineering Dirac surface states in heteroepitaxial topological crystalline insulator thin films. Nat. Nanotechnol..

[36] Springholz G, Wiesauer K (2001). Nanoscale dislocation patterning in PbTe/PbSe(001) lattice-mismatched heteroepitaxy. Phys. Rev. Lett..

[37] Walkup D (2018). Interplay of orbital effects and nanoscale strain in topological crystalline insulators. Nat. Commun..

[38] Peng R (2020). Picoscale structural insight into superconductivity of monolayer FeSe/SrTiO_3_. Sci. Adv..

[39] Hirth, J. P. & Lothe, J. *Theory of Dislocations* (Cambridge University Press, Cambridge, 1982).

[40] Springholz G (1997). Strain contrast in scanning tunneling microscopy imaging of subsurface dislocations in lattice-mismatched heteroepitaxy. Appl. Surf. Sci..

[41] Terao K, Kashiwagi T, Shizu T, Klemm RA, Kadowaki K (2019). Superconducting and tetragonal-to-orthorhombic transitions in single crystals of FeSe_1−x_Te_x_ (0 ≤ x ≤ 0.61). Phys. Rev. B.

[42] Chu J-H (2010). In-plane electronic anisotropy in underdoped Ba(Fe_1−x_Co_x_)_2_As_2_ revealed by partial detwinning in a magnetic field. Phys. Rev. B.

[43] Song C-L (2016). Observation of double-dome superconductivity in potassium-doped FeSe thin films. Phys. Rev. Lett..

[44] Miyata Y, Nakayama K, Sugawara K, Sato T, Takahashi T (2015). High-temperature superconductivity in potassium-coated multilayer FeSe thin films. Nat. Mater..

