# Supplementary material for: Nanoscale decoupling of electronic nematicity and structural anisotropy in FeSe thin films
Source: Nat Commun. 2021 Jan 4;12:10. doi: 10.1038/s41467-020-20150-y (PMC7782804; doi:10.1038/s41467-020-20150-y)
Supplement: Supplementary file 1 — Supplementary Information [file 41467_2020_20150_MOESM1_ESM.pdf]

Supplementary Information

Supplementary Note 1 Estimate of the structural modulation network spacing based on lattice mismatch

The modulation lines observed in multilayer FeSe/$SrTiO_3$(001) are roughly along ⟨110⟩ orientation (the nearest-neighbor Fe-Fe direction). Assuming the film surface is a free surface with a bulk lattice constant a_f that is smaller than the substrate lattice constant a_s, a straightforward geometrical condition dictates that n times the substrate lattice constant a_s can accommodate $n+1$ times a_f. Therefore

$$n = \frac{a_f}{a_s - a_f}, \tag{1}$$

and the dislocation lines spacing is

$$l = \frac{a_f a_s}{a_s - a_f}. \tag{2}$$

Note that the dislocation lines are along ⟨110⟩ orientation. In our case, a_f and a_s in this direction are $\sqrt{2}$ of the in-plane lattice constants of FeSe and $SrTiO_3$, which are 0.377 nm and 0.39 nm, respectively. Using the formula above, l is calculated to be ∼16 nm, which is within the range of experimental dislocation network spacings observed. We note that the system exhibits small structural buckling along the c-axis, but as this does not break any in-plane symmetries, we do not expect it to play a role in determining if an electronic nematic domain would be oriented along a- or b-axis.

Supplementary Note 2 Non-dispersing charge stripes in conductance maps

In contrast to the C_2-symmetric dispersing features pinned around the impurities, the charge-ordered stripes do not show evidence of dispersion in a range of sample biases measured (Supplementary Figure 1).

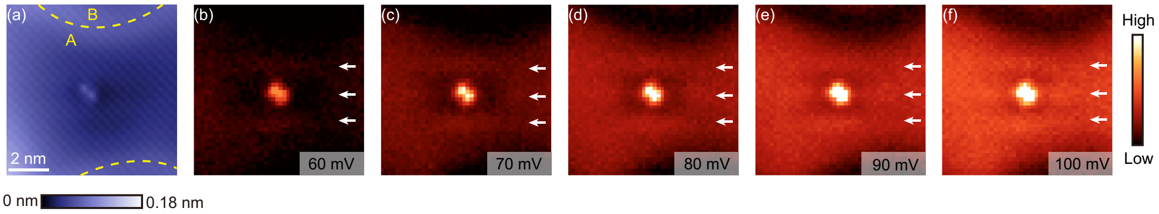

Supplementary Figure 1: (a) STM topograph over the same region as that in Supplementary Figure 2 (domain A), and (b)-(f) simultaneously acquired dI/dV maps showing the non-dispersive charge stripes. STM setup conditions: (a) I_{set} = 110 pA, V_{sample} = -100 mV; (b)-(f) V_{sample} = -100 mV, I_{set} = 110 pA, V_{exc} = 5 mV.

Supplementary Note 3 Determination of the strain tensor maps from STM topographs

An STM topograph is a quasi-periodic function $T(\mathbf{r})$ defined in a 2-dimentional real-space. A perfectly periodic atomic lattice can be expressed using a discrete Fourier series:

$$T(\mathbf{r}) = \sum_{\mathbf{g}} H_{\mathbf{g}} e^{i\mathbf{g}\cdot\mathbf{r}}, \tag{3}$$

where $\mathbf{g}$ represents reciprocal lattice vectors. A small spatially-varying distortion from this ideal lattice can be accounted for by changing $H_{\mathbf{g}}$ into $H_{\mathbf{g}} = A_{\mathbf{g}}(\mathbf{r})e^{iP(\mathbf{r})}$. The phase $P(\mathbf{r})$ is equivalent to the displacement field $\mathbf{u}(\mathbf{r})$, defined as: $-\mathbf{g}\cdot\mathbf{u}(\mathbf{r}) = P(\mathbf{r})$. Combing these, we have:

$$T(\mathbf{r}) = \sum_{\mathbf{g}} A_{\mathbf{g}} e^{i\mathbf{g}\cdot(\mathbf{r}-\mathbf{u}(\mathbf{r}))}. \tag{4}$$

With $\mathbf{u}(\mathbf{r})$ determined by the algorithm, one can apply a transformation:

$$\mathbf{r} \to \mathbf{r}' = \mathbf{r} - \mathbf{u}(\mathbf{r}) \tag{5}$$

and the topograph expressed in terms of new coordinates $\mathbf{r}'$:

$$T(\mathbf{r}') = \sum_{\mathbf{g}} A_{\mathbf{g}} e^{i\mathbf{g}\cdot\mathbf{r}'}. \tag{6}$$

will be a perfectly periodic lattice.

We determine $\mathbf{u}(\mathbf{r})$ from atomically resolved STM topographs using the Lawler-Fujita algorithm described in Ref. 1. The coarsening length scale $L = 1/\Lambda_u$, as defined in Ref. 1, is indicated in the caption of each strain analysis figure. The strain tensor $u_{ij}(\mathbf{r})$ is defined as the gradient of the displacement field $\mathbf{u}(\mathbf{r})$:

$$u_{ij}(\mathbf{r}) = \frac{1}{2}\left(\frac{\partial u_i}{\partial x_j} + \frac{\partial u_j}{\partial x_i}\right). \tag{7}$$

Empirically, certain precautions need to be taken when calculating the strain tensor from the displacement field. The real strain caused by lattice mismatch constitutes a portion of $\mathbf{u}(\mathbf{r})$, while another significant contribution comes from piezoelectric nonlinearity, thermal drift, and hysteretic effects of the STM scanner. These effects can be represented by a slowly varying "background" in $\mathbf{u}(\mathbf{r})$ [2,3], and here we apply 2nd-degree polynomial fit to remove it.

Supplementary Note 4 Bias-independence of the strain maps and data reproducibility

We show that quasi-periodic structural modulations and strain variations are intrinsically of a structural origin by showing the bias-independence of the strain maps (Supplementary Figures 2 and 4).

We also demonstrate data reproducibility on multiple samples (sample #1 in the main text and Supplementary Figure 2; sample #2 in Supplementary Figures 4 and 5). While the sample shown in the main text was capped with ~50 nm thick amorphous Se, transferred to the STM in air and then de-capped in UHV, the sample shown in Supplementary Figures 4 and 5 was grown and transferred in a vacuum suitcase to

STM, entirely in UHV conditions. Structural modulations and electronic nematicity were observed on thicker layers in both samples, indicating they are independent of the de-capping procedure.

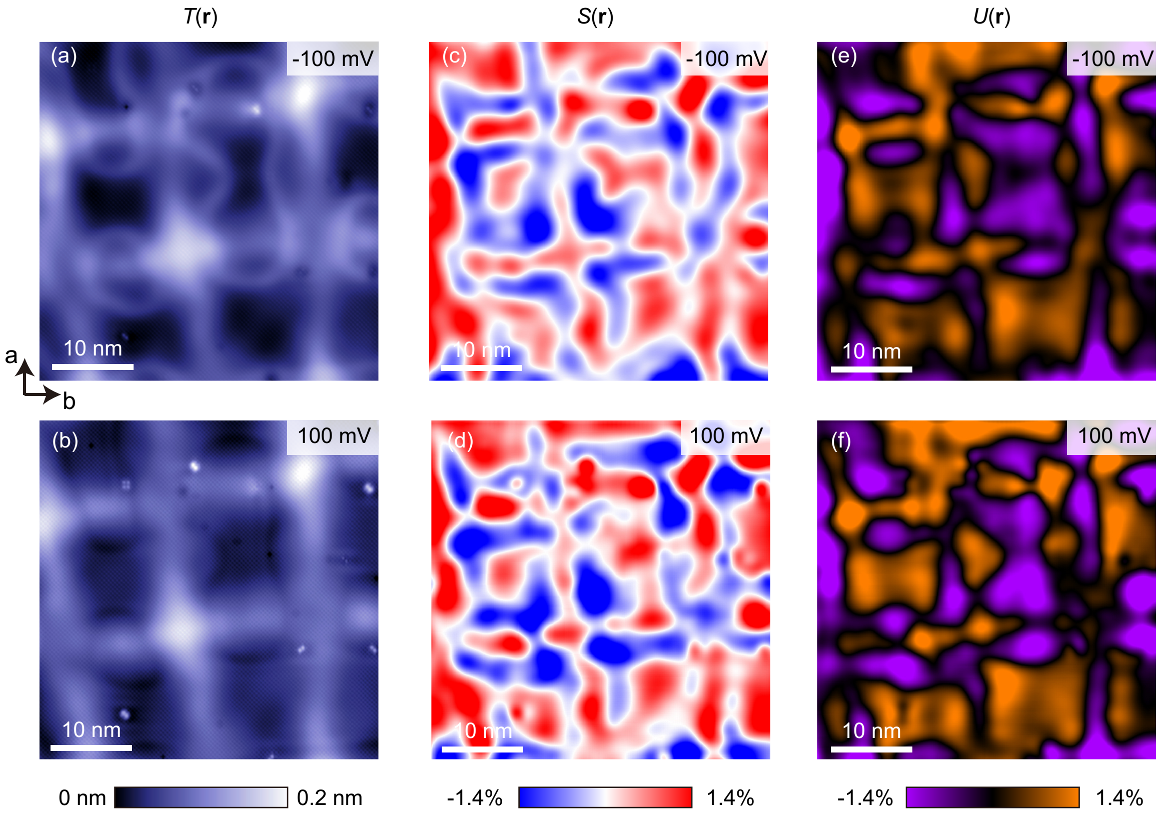

Supplementary Figure 2: (a,b) STM topographs over the same region of the sample acquired at two different STM biases, and corresponding (c,d) symmetric strain maps $S(\mathbf{r})$, and (e,f) antisymmetric strain maps $U(\mathbf{r})$. Coarsening length scale L (as defined in Supplementary Note 3) used to obtain strain tensor components in panels (c-f) was $2a$, where a is the Se-Se lattice constant. STM setup conditions: (a) I_{set} = 110 pA, V_{sample} = -100 mV; (b) I_{set} = 60 pA, V_{sample} = 100 mV.

Figure Name	FOV	Number of Pixels
Supplementary Figure 2(a)	47 nm	640 × 640
Supplementary Figure 2(b)	47 nm	896 × 896
Supplementary Figure 4(a)	33 nm	352 × 352
Supplementary Figure 4(b)	33 nm	352 × 352
Supplementary Figure 4(c)	33 nm	256 × 256
Supplementary Figure 5(a)	34 nm	256 × 256

Supplementary Table 1: Field of view (FOV) size and the number of pixels for each raw STM topograph used for strain analysis.

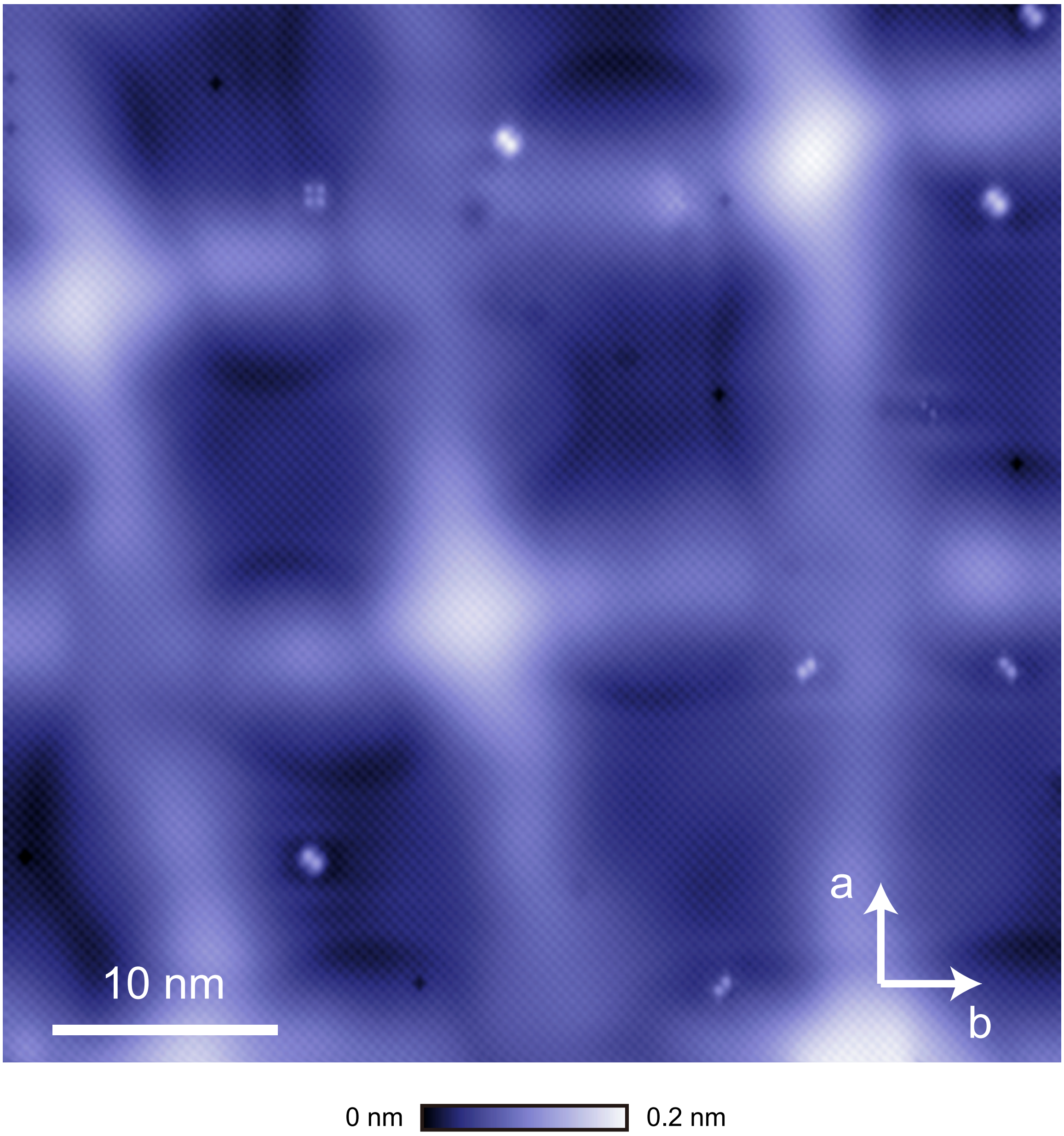

Supplementary Figure 3: A raw topograph of an area containing the region shown in Supplementary Figure 2(b). The resolution is ∼7 pixels per atom along Se-Se directions.

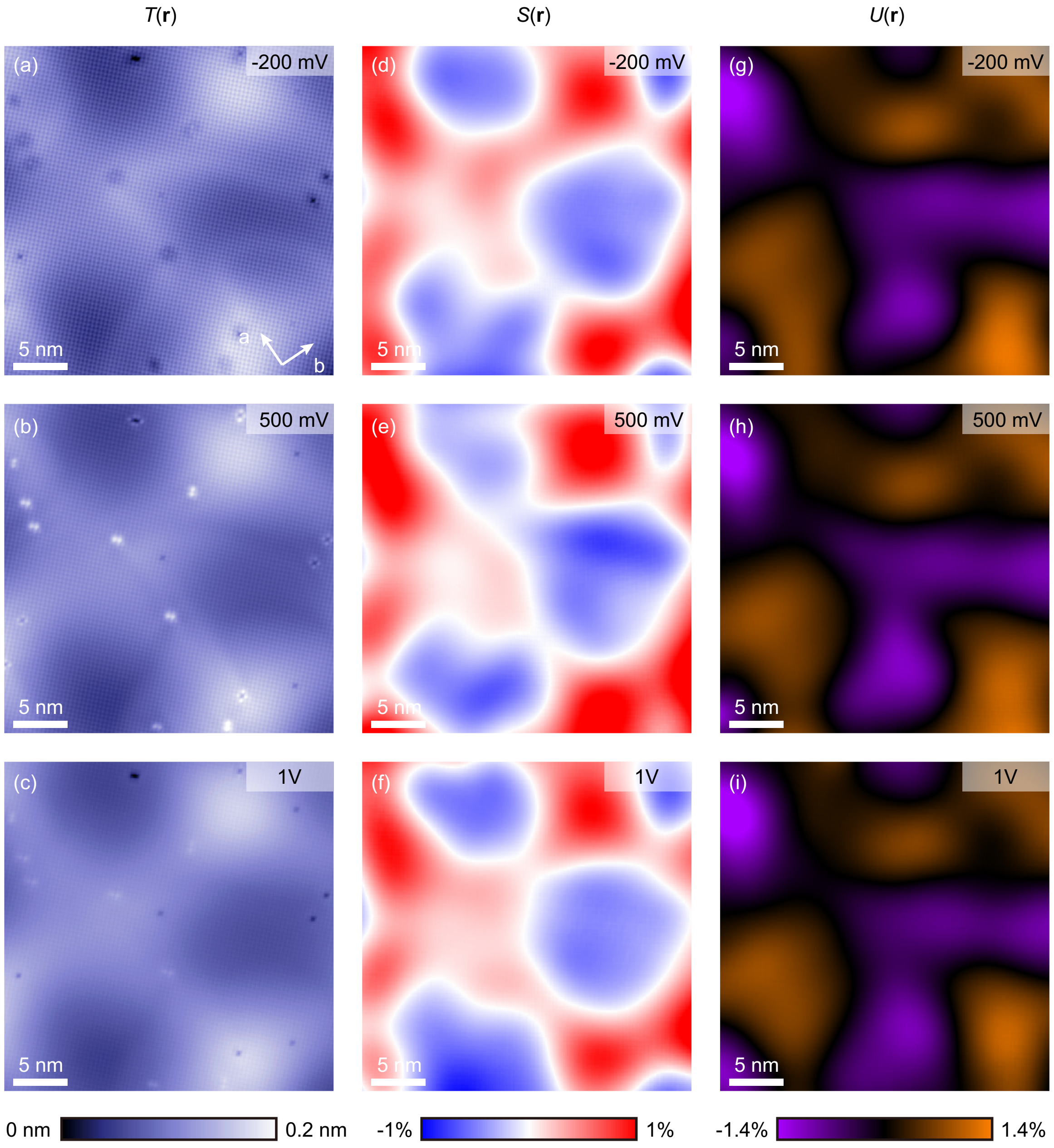

Supplementary Figure 4: (a-c) STM topographs over the same region of the sample at different bias voltages, corresponding (d-f) symmetric strain maps $S(\mathbf{r})$ and (g-i) antisymmetric strain maps $U(\mathbf{r})$ (sample 2). Coarsening length scale L (as defined in Supplementary Note 3) used to obtain strain tensor components in panels (d-i) was $5a$, where a is the Se-Se lattice constant. STM setup conditions: (a) I_{set} = 10 pA, V_{sample} = -200 mV; (b) I_{set} = 10 pA, V_{sample} = 500 mV; (c) I_{set} = 10 pA, V_{sample} = 1 V.

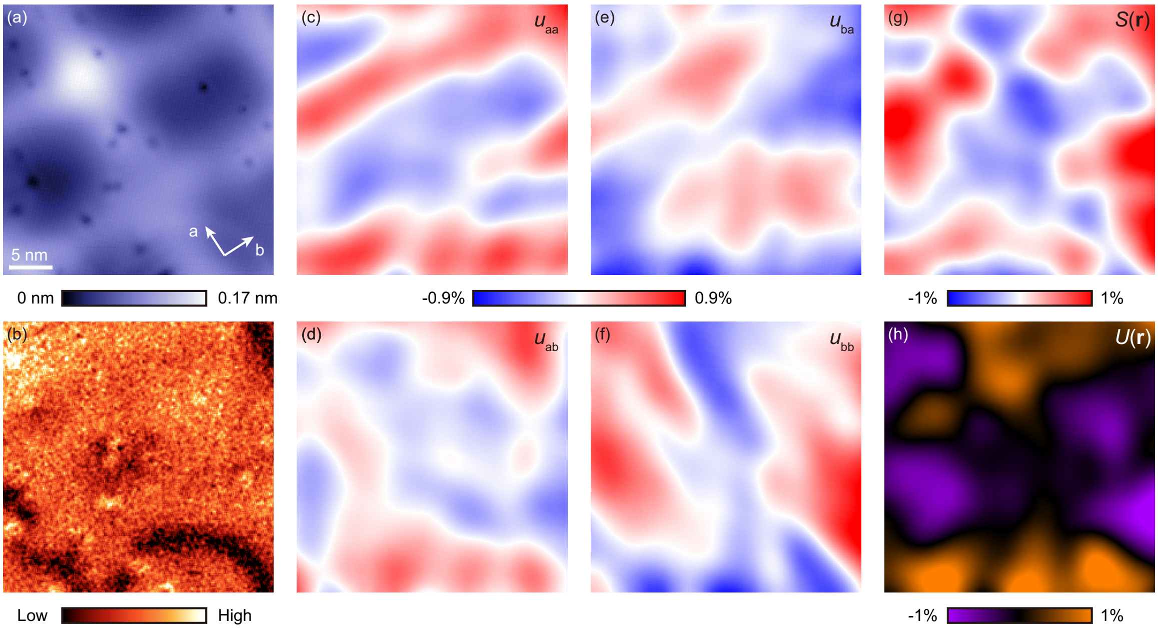

Supplementary Figure 5: (a) Atomically-resolved STM topograph (sample 2), and (b) $\mathrm{d}I/\mathrm{d}V(\mathbf{r},\ V = \text{-}300\ \mathrm{mV})$ map over the same region, showing nematic boundaries as dark lines. Strain tensor components (c) $u_{aa}(\mathbf{r})$, (d) $u_{ab}(\mathbf{r})$, (e) $u_{ba}(\mathbf{r})$, and (f) $u_{bb}(\mathbf{r})$ derived from (a). (g) Symmetric strain map $S(\mathbf{r})$. (h) Antisymmetric strain map $U(\mathbf{r})$. Coarsening length scale L (as defined in Supplementary Note 3) used to obtain strain tensor components in panels (c-h) was $5a$, where a is the Se-Se lattice constant. STM setup conditions: (a) I_{set} = 40 pA, V_{sample} = -300 mV. (b) I_{set} = 40 pA, V_{sample} = -300 mV, V_{exc} = 10 mV.

Supplementary Note 5 Determination of the surface reconstruction of $SrTiO_3(001)$ substrate

We determine the $\sqrt{13} \times \sqrt{13}$ $R33.7°$ $SrTiO_3$ surface reconstruction by a RHEED image of $SrTiO_3$ and STM topograph of 1 ML FeSe (Supplementary Figure 6(a,b)). Similar RHEED pattern has been reported in Ref. 4, where the surface reconstruction was confirmed by low energy electron diffraction (LEED) as well. On the 1 ML FeSe, we observed a stripe-like modulation. The angle between the stripes and the Se-Se lattice vector is 34.8° and the width of the stripes is $\sim\sqrt{13}a_{\text{Se-Se}}$. As the direction of Se-Se is along the same direction as Ti-Ti in $SrTiO_3$, we ascribe the stripe-like reconstruction to a $\sqrt{13} \times \sqrt{13}$ $R33.7°$ $SrTiO_3$ surface reconstruction.

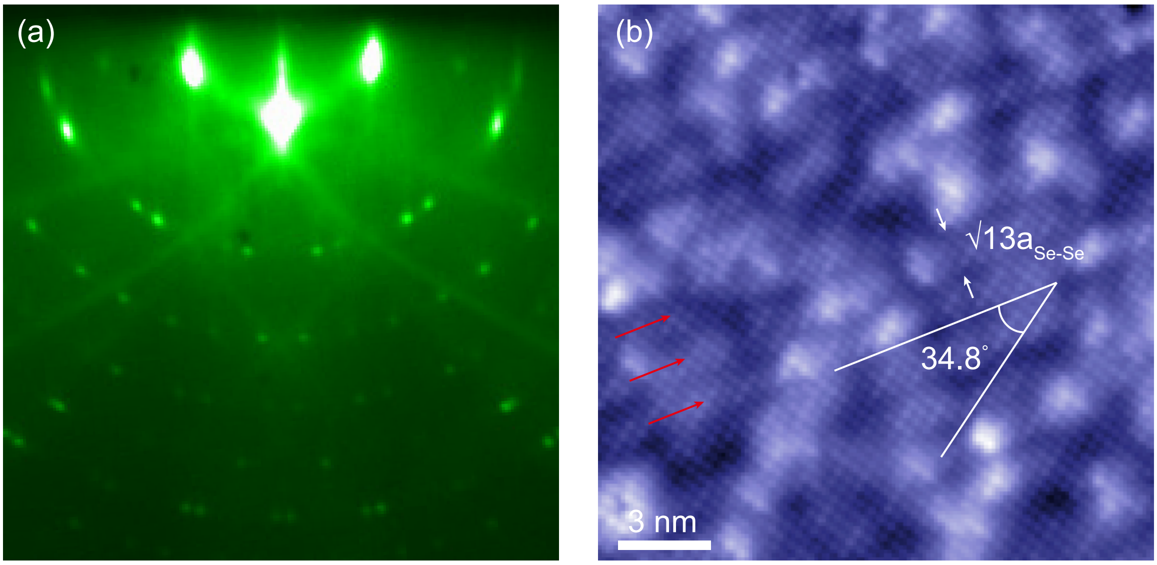

Supplementary Figure 6: (a) RHEED pattern of thermally treated $SrTiO_3(001)$ substrate showing $\sqrt{13} \times \sqrt{13}$ $R33.7°$ reconstruction. (b) STM topograph of 1 ML FeSe that shows stripe-like reconstruction. Red arrows indicate the approximate position of some of the stripes. The angle 34.8° is measured between the direction of the stripes and topmost Se-Se lattice vector. The width of the stripes is measured to be $\sim\sqrt{13}a_{\text{Se-Se}}$. RHEED was taken using a 15 keV electron gun (Staib). STM setup conditions: (b) $I_{\text{set}} = 70$ pA, $V_{\text{sample}} = 100$ mV.

Supplementary Note 6 Quasiparticle interference (QPI) imaging of 1 ML FeSe

In 1 ML FeSe, clear QPI electronic modulations can be seen in $\mathrm{d}I/\mathrm{d}V$ maps at different energies around the Fermi level (Supplementary Figure 7(a)-(d)). Fourier transforms of $\mathrm{d}I/\mathrm{d}V$ maps (Supplementary Figure 7(e)–(h)) show QPI peaks, denoted by circles at position vectors $\mathbf{q}_1$ and $\mathbf{q}_2$, which are caused by scattering between the electron pockets centered at M points of the Brillouin zone (inset in Supplementary Figure 7(i)). We also observe a superconducting gap of ∼9 meV magnitude in differential conductance spectra over the same region (Supplementary Figure 7(j)) and a wide gap-like opening observed in $\mathrm{d}I/\mathrm{d}V$ spectra acquired over a larger energy range, consistent with the gap at the Γ point (Supplementary Figure 7(k)). Both the QPI peaks and the superconducting gap are consistent with previous works [5,6] .

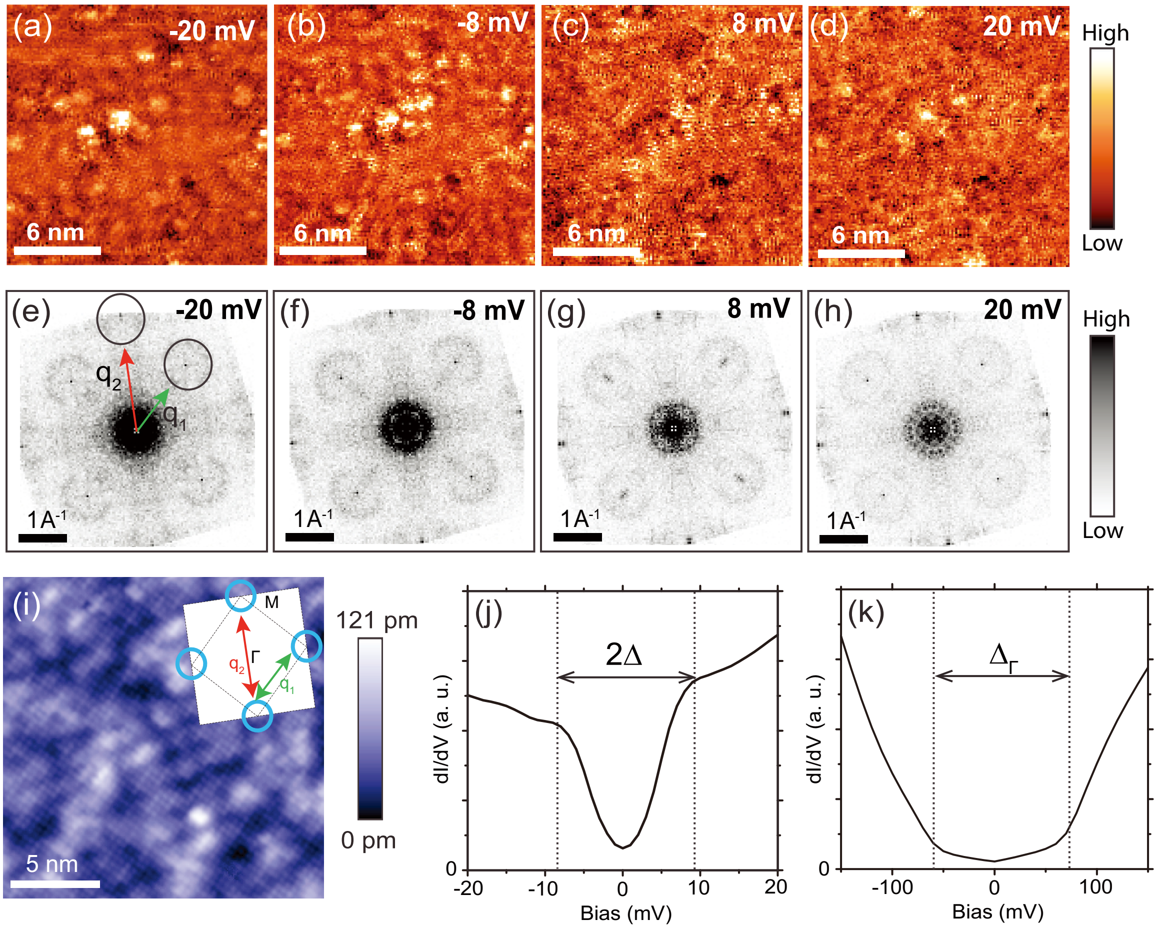

Supplementary Figure 7: (a)-(d) $\mathrm{d}I/\mathrm{d}V$ maps of 1 ML FeSe on $SrTiO_3$(001) substrate at various bias voltages. (e)-(h) corresponding Fourier transforms of respective energies of $\mathrm{d}I/\mathrm{d}V$ maps. (i) typical STM topograph of 1 ML FeSe. Inset is the schematic of the electron pockets at the corners of the 1st Brillouin zone. Scattering wavevectors $\mathbf{q}_1$ and $\mathbf{q}_2$ are denoted in (e). (j) average $\mathrm{d}I/\mathrm{d}V$ spectra showing the superconducting gap $2\Delta \sim$18 meV. (k) average spectrum showing the gap at Γ point.

Supplementary Note 7 Calculations of the theoretical strain distribution arising from the dislocation lines

We model each modulation line as a single edge dislocation at the interface of the film and the substrate. The theoretically expected strain arising from an edge dislocation can be calculated using elasticity theory [7,8]. Since FeSe film has a smaller in-plane lattice constant compared to the $SrTiO_3$(001) substrate, each edge dislocation that would form in FeSe during the growth process would consist of an extra half-plane of atoms perpendicular to the interface (Fig. 3(f)). The in-plane displacement field $u(x)$ on the surface of FeSe, caused by a dislocation line as a function of distance x from its core is predicted to be [8]:

$$u(x) = \frac{b}{\pi}\left(\frac{dx}{x^2+d^2} - \arctan\frac{x}{d}\right), \tag{8}$$

where b is the Burgers vector and d is the thickness of the film from the origin of misfit dislocation near the interface to the surface. Taking its derivative, we can obtain the theoretically predicted strain tensor component. For example, u_{xx} can be written as:

$$\frac{\mathrm{d}u(x)}{\mathrm{d}x} = -\frac{2b}{\pi d}\frac{(\frac{x}{d})^2}{((\frac{x}{d})^2+1)^2}. \tag{9}$$

Without loss of generality, strain tensor components can be determined along any vector directions, and in this work we choose the two Fe-Fe lattice directions (a- and b-axes). We calculate the theoretical u_{aa} (u_{bb}) maps as the superposition of the strain caused by 3 dislocation lines oriented roughly along the b-axis (a-axis) (Fig. 3(g,h)). We note that the calculated strain from elasticity theory is relative to the lattice constant of the undeformed substrate and consequently, it always takes negative values (Fig. 3(g-i)), which should be distinguished from the experimental strains calculated from STM topographs. However, the theoretical antisymmetric strain map can be directly compared to the experimental one since it represents the difference in u_{aa} and u_{bb}, and they show surprising resemblance (Fig. 3(j)). We emphasize that the theoretical model does not take into account any structural anisotropy due to electronic nematic state in FeSe.

Supplementary Note 8 Discussion on the absence of superconductivity at the surface of multilayer FeSe/SrTiO$_3$(001)

Some of the initial MBE growth of FeSe on graphitized SiC demonstrated that both ultrathin and thick FeSe films are superconducting, with the T_c ~8 K in thick films (comparable to that in bulk single crystals) getting reduced to ~2 K in the monolayer limit [9,10]. The reduction of T_c approaching the 2D limit can be theoretically explained by an additional boundary condition in Ginzberg-Landau free energy equation [11]. However, FeSe on $SrTiO_3$ behaves very differently. While a monolayer FeSe on $SrTiO_3$ shows a dramatic increase of T_c up to ~60-100 K, the surface of the second FeSe layer on $SrTiO_3$ is already not superconducting as shown from STM $\mathrm{d}I/\mathrm{d}V$ spectra [12,14]. Interestingly, bulk transport of thicker FeSe films ($\geq$ 2 monolayer thickness) on $SrTiO_3$ still shows superconductivity, suggesting that superconductivity originates from the interface of FeSe and $SrTiO_3$ itself, even though the topmost layer is not superconducting [12].

There may be several reasons for the absence of superconductivity at the surface of thicker FeSe films on $SrTiO_3$. First and foremost, it is possible that electronic nematicity enhanced by strain in multilayer thin films (T_N in multilayer FeSe films is ~120 K [13] compared to ~90 K in the bulk) directly competes with superconductivity. It has also been argued that insufficient charge carrier doping away from the interface may be responsible [14]. This is consistent with experiments using K surface doping, which leads to the return of superconductivity at the surface of thicker FeSe films [15]. Lastly, it is worth noting that superconducting mutilayer FeSe films on SiC mentioned above are nearly "free-standing" (based on the fact that FeSe islands grown on SiC can be moved by an STM tip, which in turn suggests a weak coupling of FeSe to the substrate, with nearly zero effective strain) [9]. On the other hand, FeSe films grown on $SrTiO_3$ are more strongly bonded to the substrate – there are no reports of moving the FeSe islands on $SrTiO_3$ and there clearly exists large spatially varying strain. So it is likely that strain indeed plays some role in explaining why thicker films are not superconducting, possibly by enhancing electronic nematicity.

Supplementary References

1. Lawler, M. J. et al. Intra-unit-cell electronic nematicity of the high-T_c copper-oxide pseudogap states. *Nature* **466**, 347 (2010).
2. Gao, S. et al. Atomic-scale strain manipulation of a charge density wave. *Proc. Natl. Acad. Sci.* **115**, 6986 (2018).
3. Walkup, D. et al. Interplay of orbital effects and nanoscale strain in topological crystalline insulators. *Nat. Commun.* **9**, 1550 (2018).
4. Zou, K. et al. Role of double TiO_2 layers at the interface of FeSe/$SrTiO_3$ superconductors. *Phys. Rev. B* **93**, 180506 (2016).
5. Liu, D. et al. Phase diagram and electron indication of high-temperature superconductivity at 65 K in single layer FeSe films. *Nat. Mater.* **12**, 605 (2013).
6. Huang, D. et al. Revealing the empty-state electronic structure of single-unit-cell FeSe/$SrTiO_3$. *Phys. Rev. Lett.* **115**, 017002 (2015)
7. Hirth, J. P. & Lothe, J. *Theory of Dislocations.* (1982).
8. Springholz, G. Strain contrast in scanning tunneling microscopy imaging of subsurface dislocations in lattice-mismatched heteroepitaxy. *Appl. Surf. Sci.* **112**, 12 (1997).
9. Song, C. et al. Molecular-beam epitaxy and robust superconductivity of stoichiometric FeSe crystalline films on bilayer graphene. *Phys. Rev. B* **84**, 020503 (2011).
10. Song, C. et al. Direct observation of nodes and twofold symmetry in FeSe superconductor. *Science* **332**, 1410 (2011).
11. Simonin, J. Surface term in the superconductive Ginzburg-Landau free energy: application to thin films. *Phys. Rev. B* **33**, 7830 (1986).
12. Wang, Q. et al. Interface-induced high-temperature superconductivity in single unit-cell FeSe films on $SrTiO_3$. *Chin. Phys. Lett.* **29**, 037402 (2012).
13. Li, W. et al. Stripes developed at the strong limit of nematicity in FeSe film. *Nat. Phys.* **13**, 957 (2017).
14. Liu, X. et al. Dichotomy of the electronic structure and superconductivity between single-layer and double-layer FeSe/$SrTiO_3$ films. *Nat. Commun.* **5**, 5047 (2014).
15. Miyata, Y. et al. High-temperature superconductivity in potassium-coated multilayer FeSe thin films. *Nat. Mater.* **14**, 775 (2015).
