# Supplementary material for: Nanoscale decoupling of electronic nematicity and structural anisotropy in FeSe thin films
Source: Nat Commun. 2021 Jan 4;12:10. doi: 10.1038/s41467-020-20150-y (PMC7782804; doi:10.1038/s41467-020-20150-y)
Supplement: Supplementary file 2 — Peer Review File [file 41467_2020_20150_MOESM2_ESM.docx]

This manuscript has been previously reviewed at another journal that is not operating a transparent peer review scheme. The manuscript was considered suitable for publication without further revisions at *Nature Communications*.
